# Integrated lipid metabolomics and proteomics analysis reveal the pathogenesis of polycystic ovary syndrome

**DOI:** 10.1186/s12967-024-05167-x

**Published:** 2024-04-17

**Authors:** Yu Qian, Yun Tong, Yaqiong Zeng, Jingyu Huang, Kailu Liu, Ying Xie, Juan Chen, Mengya Gao, Li Liu, Juan Zhao, Yanli Hong, Xiaowei Nie

**Affiliations:** Department of Reproductive Medicine, Jiangsu Province Hospital of Chinese Medicine, Affiliated Hospital of Nanjing University of Chinese Medicine, Nanjing, 210029 China

**Keywords:** Polycystic ovary syndrome, Follicular fluid, Granulosa cells, Lipid metabolomics, Proteomics, Biomarker

## Abstract

**Background:**

Polycystic ovary syndrome (PCOS) is an endocrinological and metabolic disorder that can lead to female infertility. Lipid metabolomics and proteomics are the new disciplines in systems biology aimed to discover metabolic pathway changes in diseases and diagnosis of biomarkers. This study aims to reveal the features of PCOS to explore its pathogenesis at the protein and metabolic level.

**Methods:**

We collected follicular fluid samples and granulosa cells of women with PCOS and normal women who underwent in vitro fertilization(IVF) and embryo transfer were recruited. The samples were for the lipidomic study and the proteomic study based on the latest metabolomics and proteomics research platform.

**Results:**

Lipid metabolomic analysis revealed abnormal metabolism of glycerides, glycerophospholipids, and sphingomyelin in the FF of PCOS. Differential lipids were strongly linked with the rate of high-quality embryos. In total, 144 differentially expressed proteins were screened in ovarian granulosa cells in women with PCOS compared to controls. Go functional enrichment analysis showed that differential proteins were associated with blood coagulation and lead to follicular development disorders.

**Conclusion:**

The results showed that the differential lipid metabolites and proteins in PCOS were closely related to follicle quality,which can be potential biomarkers for oocyte maturation and ART outcomes.

## Introduction

Polycystic ovary syndrome (PCOS) is the most prevalent endocrine and metabolic disorder in women, and it is also the primary cause of anovulatory infertility and hyperandrogenism [1–3]. The main clinical manifestations of the patient were oligomenorrhea, infertility, hyperandrogenism, obesity, hirsutism, acne, insulin resistance(IR) and polycystic ovarian changes under B-ultrasound. Severe metabolic disorders can cause long-term complications of PCOS,including diabetes, hyperlipidemia, cardiovascular disease and even endometrial cancer [4], which affect women's physical and psychological health. The oocytes collected from PCOS patients undergoing assisted-reproductive techniques (ART) always have poor quality, which causes a high cancelation rate and a low fertilization rate. With the pathogenesis of PCOS remaining unclear, further investigation into the etiology of PCOS and discovery of relevant biomarkers through appropriate screening, early accurate diagnosis, and effective intervention are needed to prevent long-term complications.

The most prevalent metabolic disorder in patients with PCOS is dyslipidemia. A variety of metabolic pathways involve lipids, such as steroid hormone biosynthesis, sphingolipid and fatty acid metabolism. Dyslipidemia and abnormalities in amino acid metabolism were discovered in PCOS serum and urine [5, 6]. The levels of TG, and Apo-B increased in correlation with BMI among Chinese PCOS patients [7]. Additionally, the ratios of AI, TG/HDL, and Apo-B/Apo-A were linked to specific PCOS features like insulin resistance and obesity. It has been confirmed that women with PCOS have reduced levels of sphingosine, LPE, and especially LPE (22:5) and LPC (18:2) [8]. When compared to controls, PCOS patients had significantly higher levels of lactose, stearic acid, palmitic acid, and lower levels of succinic acid in urine metabolites [9]. Sun et al. [10] found levels of bioactive lipids such as lysophosphatidylcholines (LysoPC) (16:0), phytosphingosine, LysoPC (14:0), and LysoPC (18:0) were dramatically lowered in women with PCOS, levels of free fatty acids, 3-hydroxynonanoylcarnitine carnitine, and eicosapentaenoic acid were significantly raised. The growing body of research indicates that dyslipidemia may contribute to the adverse outcome of pregnancy by eliciting an oxidative stress response.In this study,samples of follicular fluid were collected from women with PCOS and normal women who underwent IVF and embryo transfer for lipidomic analysis and granulosa cells for proteomic analysis.

Follicular fluid is composed of serum difused by local capillaries and secretions secreted by peripheral GCs, as well as exudates from plasma, primarily containing hormones, interleukins, growth factors,anti-apoptosis factors, proteins, carbohydrates, amino acids, active oxygen, and antioxidant enzymes.It provides special microenvironment for oocytes that has an effect on oocyte maturation, follicular wall rupture, fertilization, and early embryo development [11, 12]. Clear differences in a range of glycerolipid, glycerophospholipids, sphingolipids, and carboxylic acids have been found in PCOS FF [13].Granulosa cells (GCs) are specifically located around oocytes and play a critical role in oocyte maturation and ovulation [14]. GCs serve as a helpful biological model to study these challenging situations because they are also implicated in the aberrant folliculogenesis seen in diseases like PCOS. ApoA-I abundance was observed to be reduced in PCOS-afflicted women's visceral adipose tissue, entire ovarian tissue, and granulosa cells, which may have an impact on the disordered production of steroid hormones in PCOS patients [15, 16]. Since impaired oocyte quality and outcomes of IVF are associated with changes in FF and GCs components among patients with PCOS [17], the identification of the components may help to better understand and reveal potential lipid biomarkers of PCOS.

## Results

### Clinical data and ART outcomes

As shown in Table 1, we counted the mean age, body mass index (BMI), AMH, bFSH, bLH, bE2, the numbers of oocytes retrieved, MII oocytes, 2PN fertilization,number of frozen embryos,number of blastocysts,frozen embryo rate, blastocyst rate and high-quality embryos of the participants.

**Table 1:** The demographic and clinical characteristics of patients with PCOS and CON

	CON	PCOS	P
Age, years	30.38 ± 3.02	27.13 ± 5.74	0.178
BMI, kg/m2	20.85 ± 1.79	22.42 ± 2.95	0.219
Basal FSH,mIU/mL	6.78 ± 1.35	6.1 ± 1.26	0.311
Basal LH,mIU/mL	5.92 ± 3.33	13.35 ± 6.9	0.016
LH/FSH	0.88 ± 0.46	2.19 ± 1.0	0.008
Basal E2,nmol/L	47.88 ± 29.39	51.88 ± 31.14	0.795
AMH, ng/mL	5.46 ± 1.65	11.02 ± 4.48	0.009
TT, nmol/L	40.4 ± 10.61	76.92 ± 29.66	0.01
No. of oocytes retrieved	13.13 ± 6.85	18.75 ± 5.85	0.099
No. of MII oocyte	10.63 ± 6.91	16 ± 5.55	0.108
Nuclear maturation (MII) rate	0.76 ± 0.16	0.86 ± 0.17	0.242
2PN	8.75 ± 5.92	13.38 ± 5.95	0.142
2PN Fertilization rate	0.84 ± 0.2	0.83 ± 0.16	0.869
Number of frozen embryos	2.43 ± 0.79	2.33 ± 0.82	0.837
Number of blastocysts	5.4 ± 5.13	5.88 ± 4.88	0.814
Frozen embryo rate	0.59 ± 0.39	0.32 ± 0.23	0.077
Blastocyst rate	0.41 ± 0.39	0.68 ± 0.23	0.077
High-quality embryo	5.5 ± 3.89	7.63 ± 3.81	0.289
High-quality embryo rate	0.7 ± 0.24	0.59 ± 0.19	0.342
Pregnancy rate	0.625	0.75	0.000

Data are presented as mean ± SD. a Compared with the CON group. *BMI *body mass index; *bFSH *basic follicle-stimulating hormone, *bLH* basic luteinizing hormone; *bE2* basic estrogen, *AMH* Anti-Mullerian hormone; *TT* total testosterone, *MII* metaphase II.Comparison was done by unpaired t test. P < 0.05 is statistically significant

Age, BMI, bFSH, bE2, No. of retrieved oocytes, MII oocytes, fertilization,number of frozen embryos,number of blastocysts,frozen embryo rate, blastocyst rate and high-quality embryo levels were not significantly different from the CON group (P > 0.05). However, when compared to the CON group, the PCOS group's bLH, LH/FSH, and TT levels were considerably greater (P < 0.05). The pregnancy rate was slightly lower in the PCOS group than in the CON group.

### Multivariate analysis of lipid metabolites

#### Multivariate analysis of metabolites

**Fig. 1:**
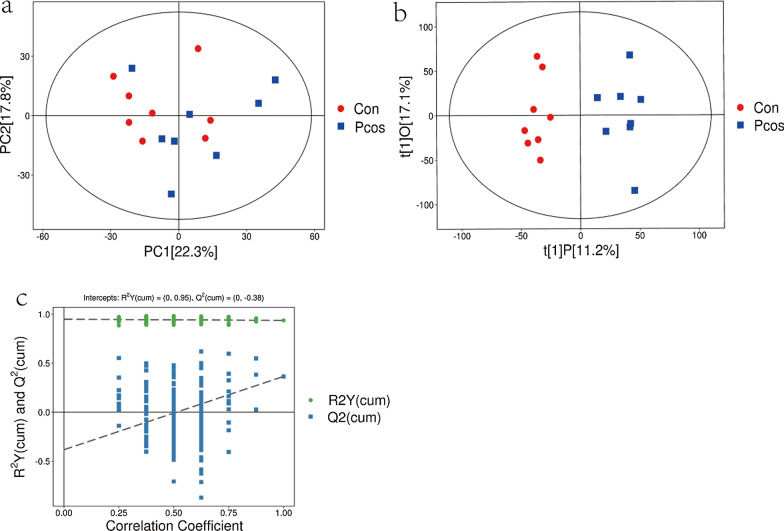
PCA score plots, OPLS-DA score plots, and corresponding validation plot of OPLS-DA results derived from FF metabolomics profiles comparing PCOS and CON.a:The PCA score scatter plot of all samples;b:The OPLS-DA score plot;c: The permutation test results of OPLS-DA model(red squares represent the control group and blue triangles represent the PCOS group).PCA: Principal component analysis;OPLS-DA:orthogonal projections to latent structures- discriminant analysis;PCOS: Polycystic ovary syndrome

16 experimental samples and 4 quality control (QC) samples made up the original data. To reduce the effect of detection system error on the results, so that the results can better highlight the biology significance, we carried out a series of preparation and collation of the original data. It mainly includes the following steps: deviation value filtering, missing value filtering, missing value imputation and data normalization. After the data had been preprocessed, SIMCA software (V16.0.2, Sartorius Stedim Data Analytics AB, Umea, Sweden) was used to analyze the data.The FF metabolic profiles were then analyzed by principal component analysis (PCA) and OPLS-DA model analysis to reflect the degree of difference between groups. Figure 1a displays the PCA score scatter plot for all samples. According to Hotelling's T squared ellipse, all samples were distributed within the 95% confidence interval. The OPLS-DA score plot results revealed that the PCOS and CON groups had significantly different lipid metabolites in FF(Fig. 1b). According to the OPLS-DA model's permutation test findings, the R2Y(cum) and Q2(cum) values of the PCOS group vs the Con group were 0.95 and -0.38, respectively (Fig. 1c). The results showed that the OPLS-DA model did not overfit and had a high predictive ability, which was suitable for subsequent optimization analysis.

#### Significant lipid differential metabolite analysis between CON and PCOS group

**Fig. 2:**
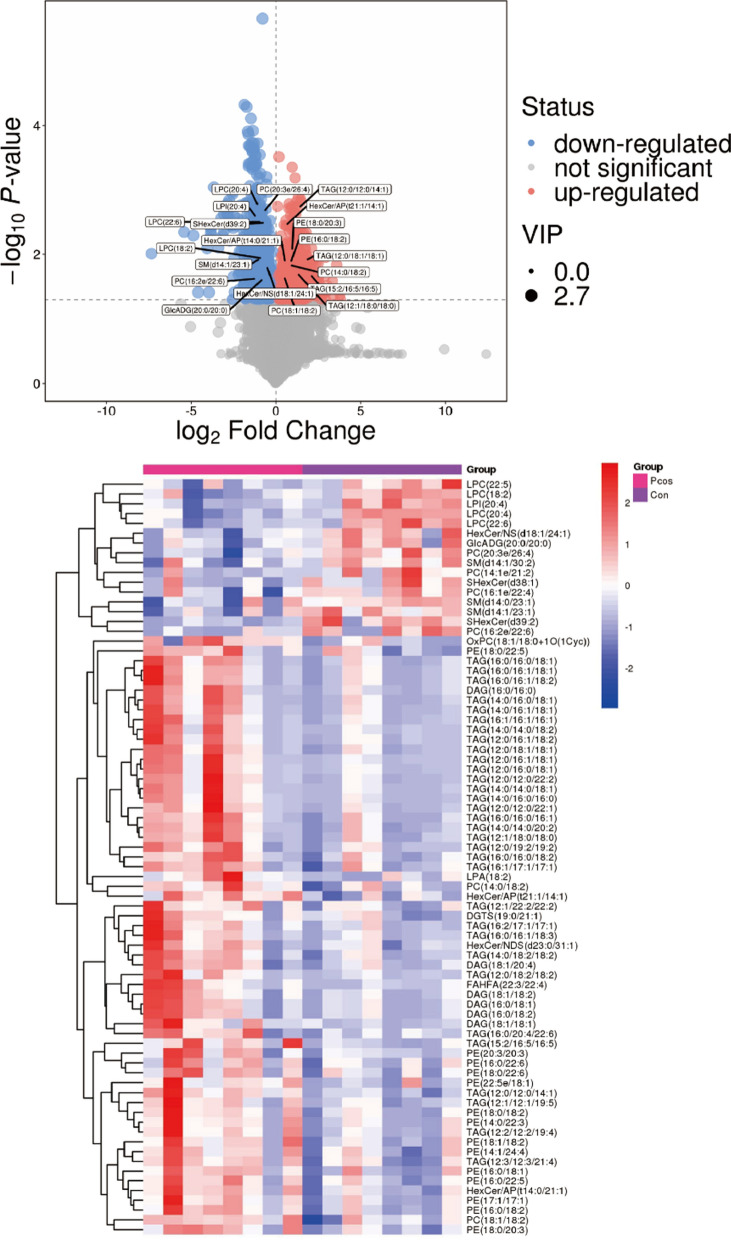
ldentification of the differential metabolomics profiles of FF between PCOS and CON patients based on a volcano plot and hierarchical clustering analysis. a. Volcano plot, down-requlated and up-regulatedmetabolites in PCOS compared to CON are marked in blue and red, respectively. The -axis represents the log2 fold change of metabolites, while the Y-axis represents the fold change of the -log10 P value determined by the Student's t-test The variable importance in the projection (VIP) value is represented by the dot size. b. Heatmap of the hierarchical clustering analysis.There are 77 distinct metabolites presented

In this study, 145 FF metabolites in the OPLS-DA analysis (VIP values > 1) and the univariate analysis (P-values < 0.05) were detected between PCOS and CON groups (Table 2). Differential lipid metabolites were represented by volcano plots (Fig. 2a) and the elevated and downregulated metabolites in each group were distinguished by a heatmap (Fig. 2b). Most lipid metabolites were increased in the FF from patients PCOS group compared to the CON group: levels of triacylglycerol(TAG), Phosphatidylethanolamine(PE), Diacylglycerol(DAG) and Hexosylceramide alpha-hydroxy fatty acid-phytospingosine(HexCer-AP) were significantly higher in PCOS FF. Whereas, when comparison to the CON group, PCOS FF had significantly lower levels of Lysophophatidylcholine(LPC), Sphingomyelin(SM), SulfurHexosylceramide hydroxyfatty acid(SHexCer) and Phosphatidylcholine(PC). Changes in other compound classes were also detected, such as Phosphatidylinositol(PI), Diacylglyceryl trimethylhomoserine (DGTS), glucuronosyldiacylglycerol(GlcADG) and etc.

**Table 2:** List of the 144 differential expressed proteins in PCOS patients’ GCs

protein	gene	gene_id	Ctrl	PCOS	FC	log2FC	p.value	adj.Pval
Q15526	SURF1	6834	284,185	173,470	− 1.64	− 0.71	0.000	0.002
Q9Y2Q3	GSTK1	373,156	1,174,341	641,119	− 1.83	− 0.87	0.000	0.003
O43676	NDUFB3	4709	400,938	238,907	− 1.68	− 0.75	0.000	0.003
Q12913	PTPRJ	5795	119,016	42,470	− 2.80	− 1.49	0.000	0.003
O95298	NDUFC2	4718	619,014	357,023	-1.73	-0.79	0.000	0.003
P09488	GSTM1	2944	1,718,348	1,042,070	-1.65	-0.72	0.000	0.003
P63218	GNG5	2787	1,576,490	763,188	− 2.07	− 1.05	0.000	0.003
O75251	NDUFS7	374,291	1,374,515	742,538	− 1.85	− 0.89	0.000	0.003
P02790	HPX	3263	4,250,366	6,880,411	1.62	0.69	0.000	0.003
P25311	AZGP1	563	751,209	1,195,863	1.59	0.67	0.000	0.003
P30837	ALDH1B1	219	2,173,243	1,349,944	− 1.61	− -0.69	0.000	0.003
Q00266	MAT1A	4143	230,553	351,976	1.53	0.61	0.000	0.003
O14949	UQCRQ	27,089	803,205	488,817	− 1.64	− 0.72	0.000	0.003
P02774	GC	2638	3,965,445	5,962,874	1.50	0.59	0.000	0.003
P00738	HP	3240	7,379,939	13,645,033	1.85	0.89	0.000	0.003
P04217	A1BG	1	1,406,796	2,258,651	1.61	0.68	0.000	0.003
Q96PQ0	SORCS2	57,537	384,503	597,165	1.55	0.64	0.000	0.003
P01889	HLA-B	3106	667,757	380,852	− 1.75	− 0.81	0.000	0.003
Q9UKD2	MRTO4	51,154	1,137,548	716,754	− 1.59	− 0.67	0.000	0.003
P51398	DAP3	7818	280,057	186,622	− 1.50	− 0.59	0.000	0.003
P0DPK3	NOTCH2NLB	-	37,072	96,710	2.61	1.38	0.000	0.003
P29508	SERPINB3	6317	310,452	104,771	− 2.96	− 1.57	0.000	0.003
Q9NR31	SAR1A	56,681	3,426,034	2,244,801	− 1.53	− 0.61	0.000	0.003
Q9HD33	MRPL47	57,129	188,825	111,188	− 1.70	− 0.76	0.000	0.003
P04259	KRT6B	3854	540,222	128,236	− 4.21	− 2.07	0.000	0.003
P28161	GSTM2	2946	137,780	72,088	− 1.91	− 0.93	0.000	0.003
P03923	MT-ND6	4541	35,932	20,316	− 1.77	− 0.82	0.000	0.003
Q9H0C2	SLC25A31	83,447	245,346	384,926	1.57	0.65	0.000	0.003
P37235	HPCAL1	3241	361,566	229,922	− 1.57	− 0.65	0.000	0.004
P62834	RAP1A	5906	233,369	153,846	− 1.52	− 0.60	0.000	0.004
O75339	CILP	8483	131,477	209,256	1.59	0.67	0.000	0.004
Q9GZT4	SRR	63,826	136,695	78,395	-1.74	-0.80	0.000	0.004
O60239	SH3BP5	9467	447,227	679,200	1.52	0.60	0.000	0.004
P11511	CYP19A1	1588	9,173,743	18,579,304	2.03	1.02	0.000	0.004
P08514	ITGA2B	3674	215,393	333,968	1.55	0.63	0.000	0.004
P53701	HCCS	3052	338,315	213,073	− 1.59	− 0.67	0.000	0.004
Q96NB2	SFXN2	118,980	79,746	48,362	− 1.65	− 0.72	0.000	0.004
Q9H9B4	SFXN1	94,081	1,452,170	896,065	− 1.62	− 0.70	0.000	0.005
O95810	CAVIN2	8436	66,812	104,678	1.57	0.65	0.000	0.005
Q9H936	SLC25A22	79,751	897,701	589,248	− 1.52	− 0.61	0.000	0.005
Q9P032	NDUFAF4	29,078	1,149,556	702,973	− 1.64	− 0.71	0.000	0.005
P33947	KDELR2	11,014	296,115	183,010	− 1.62	− 0.69	0.000	0.005
Q9Y250	LZTS1	11,178	263,805	149,676	− 1.76	− 0.82	0.000	0.005
Q14134	TRIM29	23,650	284,192	100,645	− 2.82	− 1.50	0.000	0.005
Q92954	PRG4	10,216	74,379	122,829	1.65	0.72	0.000	0.005
P08567	PLEK	5341	334,199	536,897	1.61	0.68	0.000	0.005
P01911	HLA-DRB1	3123	228,205	141,967	− 1.61	− 0.68	0.000	0.006
P12273	PIP	5304	25,201	5,741	− 4.39	− 2.13	0.000	0.006
P29373	CRABP2	1382	157,270	81,808	− 1.92	− 0.94	0.000	0.006
Q96BW9	TAMM41	132,001	55,221	16,690	− 3.31	− 1.73	0.000	0.006
Q8NGY0	OR10X1	128,367	108,409	19,110	− 5.67	− 2.50	0.000	0.006
Q9NQ66	PLCB1	23,236	218,829	121,685	− 1.80	− 0.85	0.000	0.006
Q9UDW1	UQCR10	29,796	236,050	124,516	− 1.90	− 0.92	0.000	0.006
O75964	ATP5MG	10,632	1,037,671	627,542	− 1.65	− 0.73	0.000	0.006
Q8IYA8	IHO1	339,834	219,200	47,890	− 4.58	− 2.19	0.000	0.006
Q96C55	ZNF524	147,807	19,907	32,877	1.65	0.72	0.000	0.006
P62244	RPS15A	6210	8,620,369	5,315,558	− 1.62	− 0.70	0.000	0.006
P02750	LRG1	116,844	502,437	764,431	1.52	0.61	0.000	0.007
P14923	JUP	3728	699,684	355,553	− 1.97	− 0.98	0.000	0.007
O94788	ALDH1A2	8854	221,934	142,853	− 1.55	− 0.64	0.000	0.007
Q9Y3D7	PAM16	51,025	711,534	315,270	− 2.26	− 1.17	0.000	0.007
Q9BXY0	MAK16	84,549	35,784	18,624	− 1.92	− 0.94	0.000	0.008
Q9BUR5	APOO	79,135	667,855	353,501	− 1.89	− 0.92	0.000	0.008
O95707	POP4	10,775	14,068	5,052	− 2.78	− 1.48	0.000	0.008
P00167	CYB5A	1528	1,282,803	735,534	− 1.74	− 0.80	0.001	0.008
Q9Y2Z4	YARS2	51,067	241,251	155,613	− 1.55	− 0.63	0.001	0.008
O95388	CCN4	8840	51,952	83,724	1.61	0.69	0.001	0.008
Q14CS0	UBXN2B	137,886	31,015	47,772	1.54	0.62	0.001	0.009
P46782	RPS5	6193	4,392,982	2,690,597	− 1.63	− 0.71	0.001	0.009
P17152	TMEM11	8834	642,886	416,122	− 1.54	− 0.63	0.001	0.009
P02795	MT2A	4502	19,503,859	29,591,086	1.52	0.60	0.001	0.009
Q9Y241	HIGD1A	25,994	169,400	101,036	− 1.68	− 0.75	0.001	0.009
P10909	CLU	1191	4,548,982	7,925,039	1.74	0.80	0.001	0.009
O00534	VWA5A	4013	9,880	16,060	1.63	0.70	0.001	0.009
Q9BTY2	FUCA2	2519	81,811	51,213	− 1.60	− 0.68	0.001	0.009
P19256	CD58	965	29,751	17,925	− 1.66	− 0.73	0.001	0.010
Q9GZT6	CCDC90B	60,492	51,309	23,390	− 2.19	− 1.13	0.001	0.010
P69892	HBG2	3048	120,179	70,954	− 1.69	− 0.76	0.001	0.010
O75438	NDUFB1	4707	223,826	139,189	− 1.61	− 0.69	0.001	0.010
Q96IX5	ATP5MK	84,833	360,976	208,888	− 1.73	− 0.79	0.001	0.010
P15880	RPS2	6187	6,348,391	3,722,541	− 1.71	− 0.77	0.001	0.010
P02654	APOC1	341	860,143	466,404	− 1.84	− 0.88	0.001	0.010
Q9UBG3	CRNN	49,860	76,918	24,136	− 3.19	− 1.67	0.001	0.011
P46781	RPS9	6203	5,215,220	2,227,525	− 2.34	− 1.23	0.001	0.011
P06703	S100A6	6277	1,201,162	771,837	− 1.56	− 0.64	0.001	0.012
Q8N4H5	TOMM5	401,505	1,142,248	738,886	− 1.55	− 0.63	0.001	0.012
P82930	MRPS34	65,993	121,804	65,432	− 1.86	− 0.90	0.001	0.012
P53675	CLTCL1	8218	87,078	58,043	− 1.50	− 0.59	0.001	0.012
Q5VW38	GPR107	57,720	485,922	746,582	1.54	0.62	0.001	0.012
Q5VW32	BROX	148,362	577,561	372,850	− 1.55	− 0.63	0.002	0.013
Q6DKI1	RPL7L1	285,855	149,708	70,407	− 2.13	− 1.09	0.002	0.014
P02766	TTR	7276	238,544	383,317	1.61	0.68	0.002	0.015
Q8TF42	UBASH3B	84,959	143,903	219,528	1.53	0.61	0.002	0.015
P32969	RPL9	6133	5,202,187	3,095,939	− 1.68	− 0.75	0.002	0.015
O43677	NDUFC1	4717	60,106	28,897	− 2.08	− 1.06	0.002	0.015
Q6P2S7	TTC41P	–	603,584	365,283	− 1.65	− 0.72	0.002	0.016
Q30154	HLA-DRB5	3127	44,004	11,147	− 3.95	− 1.98	0.002	0.016
Q86VV8	RTTN	25,914	311,361	196,196	− 1.59	− 0.67	0.002	0.016
P00403	MT-CO2	4513	1,399,412	886,732	− 1.58	− 0.66	0.002	0.017
Q9BRU2	TCEAL7	56,849	20,218	32,996	1.63	0.71	0.003	0.017
O96015	DNAL4	10,126	21,608	12,550	− 1.72	− 0.78	0.003	0.017
Q9UM19	HPCAL4	51,440	76,954	36,512	− 2.11	− 1.08	0.003	0.018
Q9UMX5	NENF	29,937	339,207	222,907	− 1.52	− 0.61	0.003	0.019
Q5VWG9	TAF3	83,860	11,725	18,826	1.61	0.68	0.003	0.019
Q8N442	GUF1	60,558	75,978	48,778	− 1.56	− 0.64	0.003	0.019
Q4KMQ1	TPRN	286,262	5,816	9,197	1.58	0.66	0.003	0.020
Q9Y5U9	IER3IP1	51,124	113,646	61,235	− 1.86	− 0.89	0.003	0.020
Q5U3C3	TMEM164	84,187	44,863	25,819	− 1.74	− 0.80	0.004	0.021
Q5TGZ0	MICOS10	440,574	743,045	390,926	− 1.90	− 0.93	0.005	0.026
Q96ND0	FAM210A	125,228	282,317	179,501	− 1.57	− 0.65	0.005	0.026
Q96I45	TMEM141	85,014	115,039	70,544	− 1.63	− 0.71	0.006	0.027
Q6S8J3	POTEE	445,582	493,731	167,770	− 2.94	− 1.56	0.006	0.027
Q86SE9	PCGF5	84,333	22,535	40,089	1.78	0.83	0.006	0.029
Q969U7	PSMG2	56,984	326,118	183,555	− 1.78	− 0.83	0.007	0.030
Q8NI22	MCFD2	90,411	87,857	55,515	− 1.58	− 0.66	0.007	0.031
P63167	DYNLL1	8655	2,081,999	1,351,996	− 1.54	− 0.62	0.009	0.035
P07108	DBI	1622	1,989,043	1,183,131	− 1.68	− 0.75	0.010	0.036
Q96EX1	SMIM12	113,444	85,648	29,536	− 2.90	− 1.54	0.012	0.040
P04080	CSTB	1476	509,128	312,837	− 1.63	− 0.70	0.012	0.041
Q9NX55	HYPK	25,764	89,531	136,144	1.52	0.60	0.012	0.041
P15954	COX7C	1350	1,061,237	619,515	− 1.71	− 0.78	0.012	0.042
Q9NX47	MARCHF5	54,708	50,287	31,896	− 1.58	− 0.66	0.015	0.046
Q15528	MED22	6837	610,344	405,677	− 1.50	− 0.59	0.016	0.049
P60602	ROMO1	140,823	118,431	78,365	− 1.51	− 0.60	0.016	0.049
P62805	H4C1	121,504	62,707,994	29,548,173	− 2.12	− 1.09	0.017	0.052
P20962	PTMS	5763	926,341	1,462,639	1.58	0.66	0.018	0.053
P54727	RAD23B	5887	2,645,799	1,703,085	− 1.55	− 0.64	0.019	0.054
Q13405	MRPL49	740	42,753	28,458	− 1.50	− 0.59	0.020	0.056
P00739	HPR	3250	5,937	10,998	1.85	0.89	0.020	0.057
Q15329	E2F5	1875	56,906	205,274	3.61	1.85	0.020	0.057
Q96MR6	CFAP57	149,465	32,510	49,101	1.51	0.59	0.022	0.061
Q99653	CHP1	11,261	688,597	422,414	− 1.63	− 0.71	0.023	0.062
O00483	NDUFA4	4697	2,330,413	1,102,940	− 2.11	− 1.08	0.024	0.064
O15069	NACAD	23,148	3,683	5,718	1.55	0.63	0.024	0.064
P50150	GNG4	2786	12,654	8,096	− 1.56	− 0.64	0.024	0.064
Q13835	PKP1	5317	739,970	221,000	− 3.35	− 1.74	0.024	0.064
O00292	LEFTY2	7044	1,667	3,516	2.11	1.08	0.025	0.066
Q96A72	MAGOHB	55,110	2,809,579	1,841,289	− 1.53	− 0.61	0.025	0.067
Q9NZD4	AHSP	51,327	75,582	37,533	− 2.01	− 1.01	0.028	0.072
Q14191	WRN	7486	230,368	558,126	2.42	1.28	0.029	0.073
P30536	TSPO	706	611,183	340,994	− 1.79	− 0.84	0.032	0.077
Q02413	DSG1	1828	154,954	67,828	− 2.28	− 1.19	0.040	0.090
O60437	PPL	5493	891,734	589,101	− 1.51	− 0.60	0.048	0.102
Q8NC42	RNF149	284,996	51,948	84,835	1.63	0.71	0.049	0.105

#### Correlation analysis between lipid metabolite concentrations and clinical parameters

**Fig. 3:**
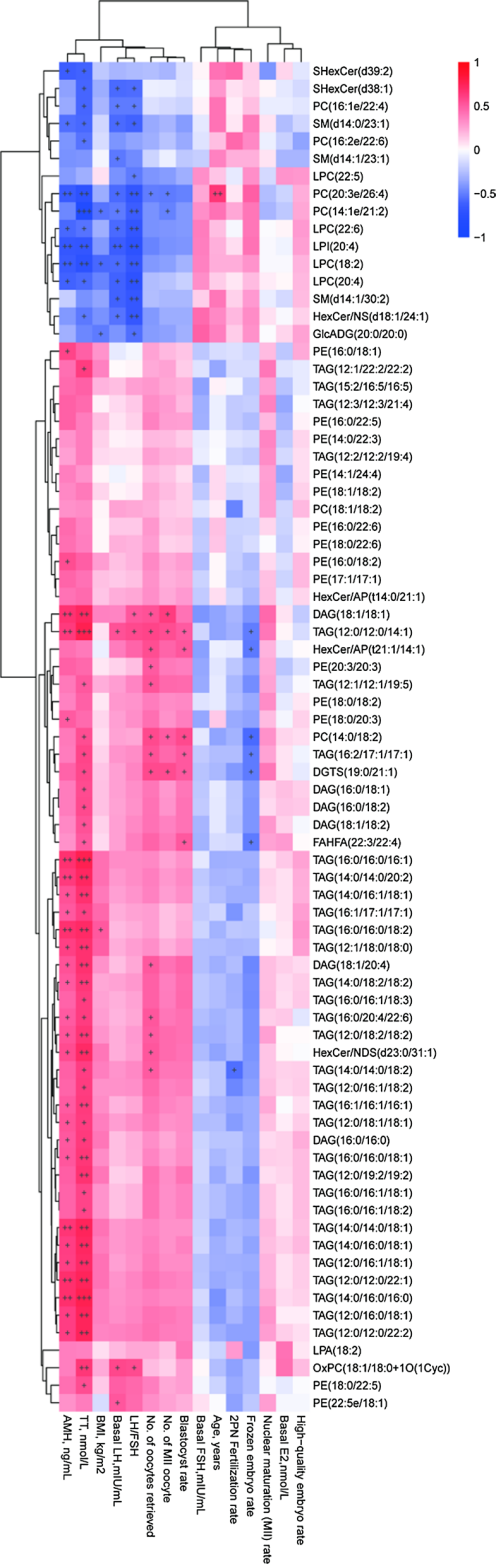
Spearman correlation analysis. The horizontal and vertical coordinates represent the metabolites and clinical indicators in this group, and the color blocks at different positions represent the correlation coefficients between metabolites and clinical indicators at corresponding positions. Red indicates positive correlation, blue indicates negative correlation, and the darker the color, the stronger the correlation. Significant associations are marked with asterisked (p < 0.05)

We performed an association analysis of differential lipid metabolites and clinical indicators between the PCOS and CON groups (Fig. 3). The results were presented by hierarchical clustering heatmap: in the PCOS versus CON group,the concentrations TAG,DAG,PE,PC and HexCer-AP were found to be positively associated with TT,AMH, No. of oocytes retrieved, MII oocytes, and fertilization (P < 0.05). Significant negative correlations were found between LPC,SM, SHexCer,PC and BMI, TT,AMH, No. of oocytes retrieved,MII oocytes and fertilization(P < 0.05). However, TAG and PE were inversely linked with age,frozen embryo rate and high-quality embryo rate.

#### Analysis of inflammatory factors and lipid metabolites

**Fig. 4:**
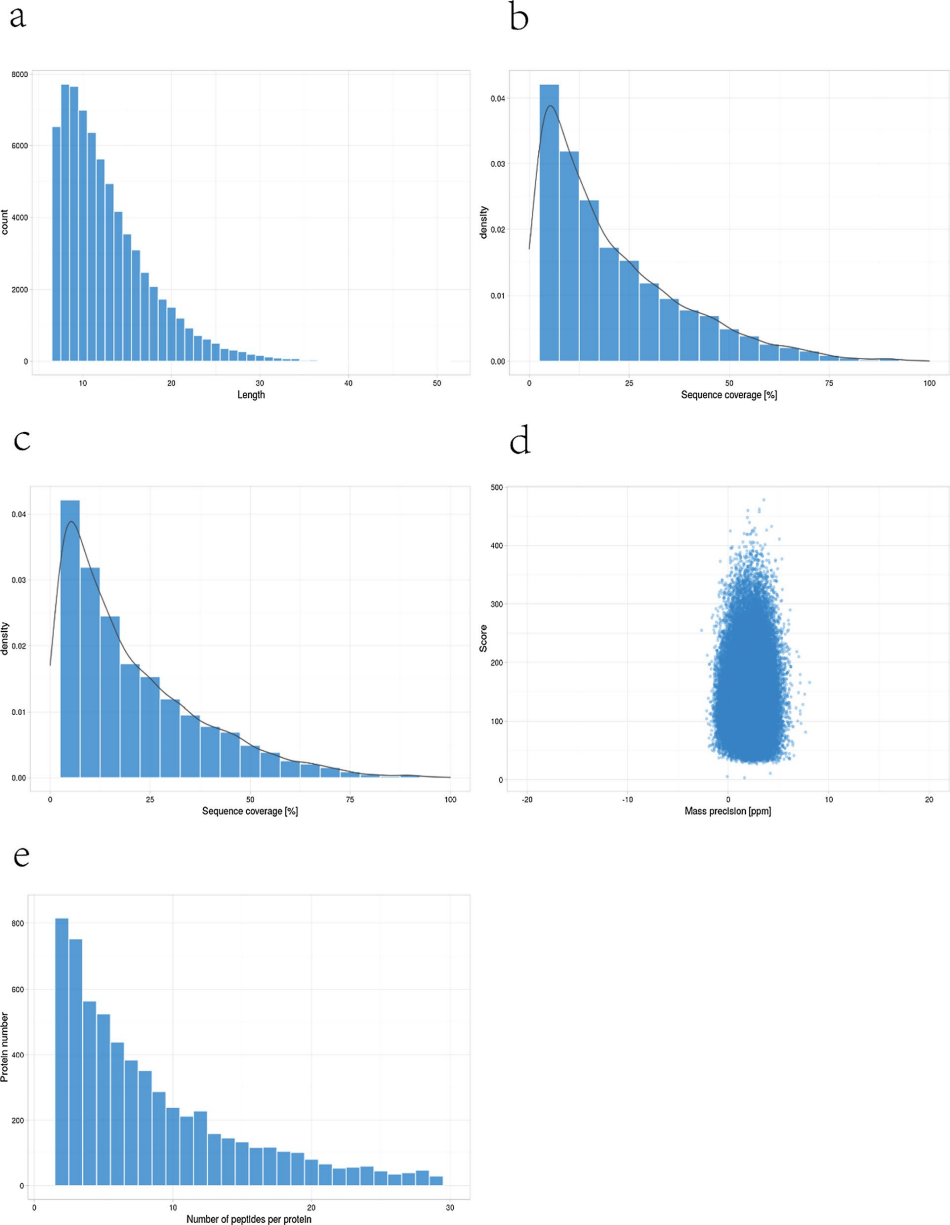
**a**, **b** FF TNF-α and IL-6 concentrationsof PCOS and CON groups;c:Correlation analysis between inflammatory factor concentrations and lipid metabolites performed by Mantel test in RStudio

The concentrations of TNF-α and IL-6 in follicular fluid of PCOS patients were significantly higher than those of the CON group(P < 0.05).In order to further explore the relationship between inflammatory factors and lipid metabolism, we selected the inflammatory factors TNF-α and IL-6, which are closely related to lipid and PCOS, for comprehensive correlation analysis.The results showed that proinflammatory variables correlated with lipid metabolites(Fig. 4c).IL-6 was positively correlated with SM(d14:0/23:1) and DAG(18:1/18:2),TNF-α was positively correlated with PC(20:3e/26:4),SM(d14:0/23:1) and LPC(18:2)(P < 0.05).

### Proteomic analysis

#### Mass spectrometry quality control detection

**Fig. 5:**
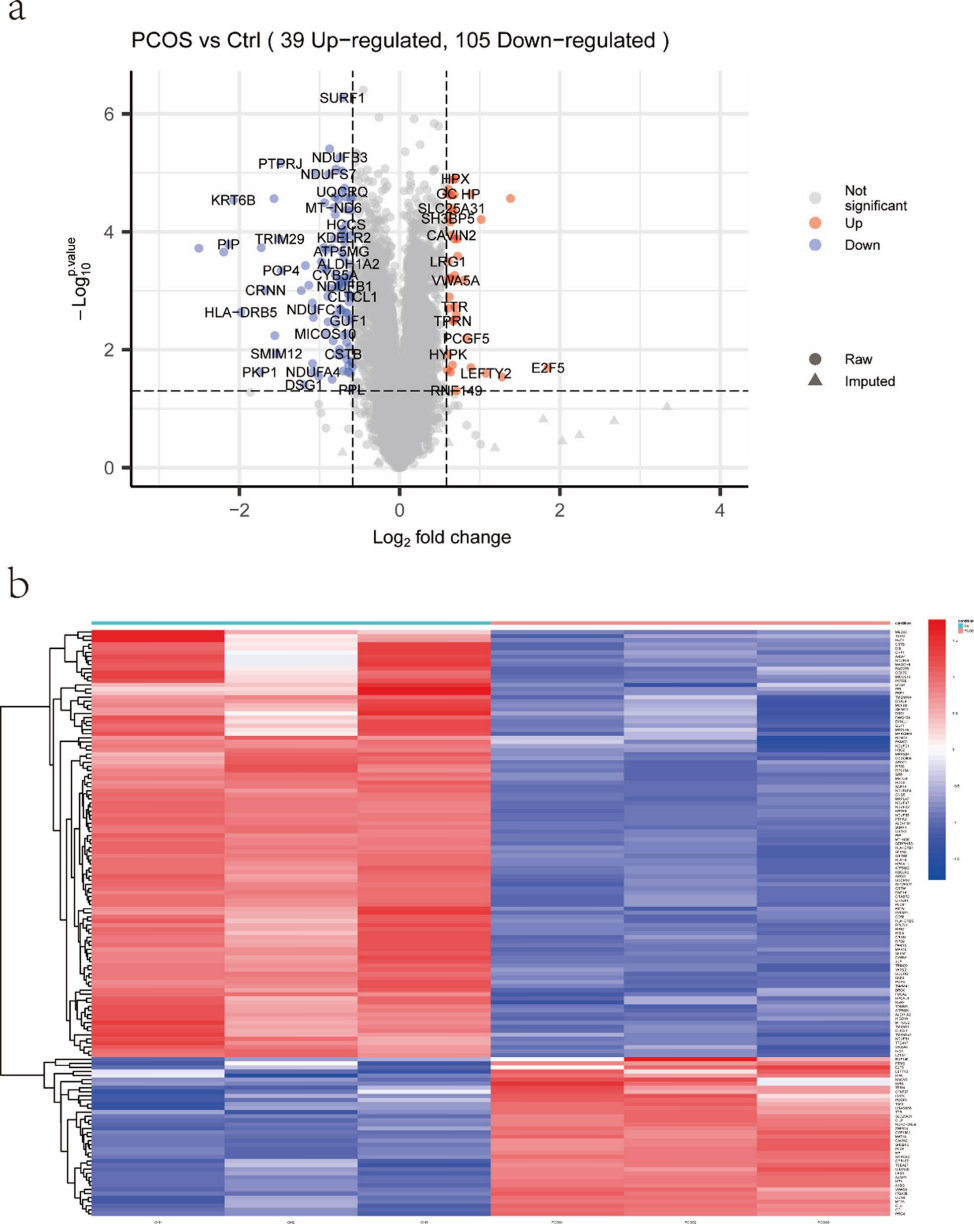
A total of 70,296 peptides and 7423 proteins were identified, of which 7326 proteins could be quantified. **a**–**c** The length distribution of peptides identified by mass spectrometry in this experiment met the quality control requirements, and the molecular weight and coverage of the experimental protein were in line with expectations. **d** The mass error of most spectra is less than 5 ppm, which accords with the high precision of orbital trap mass spectrometry. **e** Most of the proteins correspond to more than two peptides, indicating the accuracy and credibility of the quantitative results

As shown in the figure, the length distribution of peptides found by mass spectrometry in this experiment met the quality control requirements, and the molecular weight and coverage of the experimental protein were in line with expectations (Fig. 5a–c). The mass error of most spectra is less than 5 ppm, which accords with the high precision of orbital trap mass spectrometry(Fig. 5d). Most of the proteins correspond to more than two peptides, indicating the accuracy and credibility of the quantitative results(Fig. 5e).

#### Protein analysis that differs between groups

**Fig. 6:**
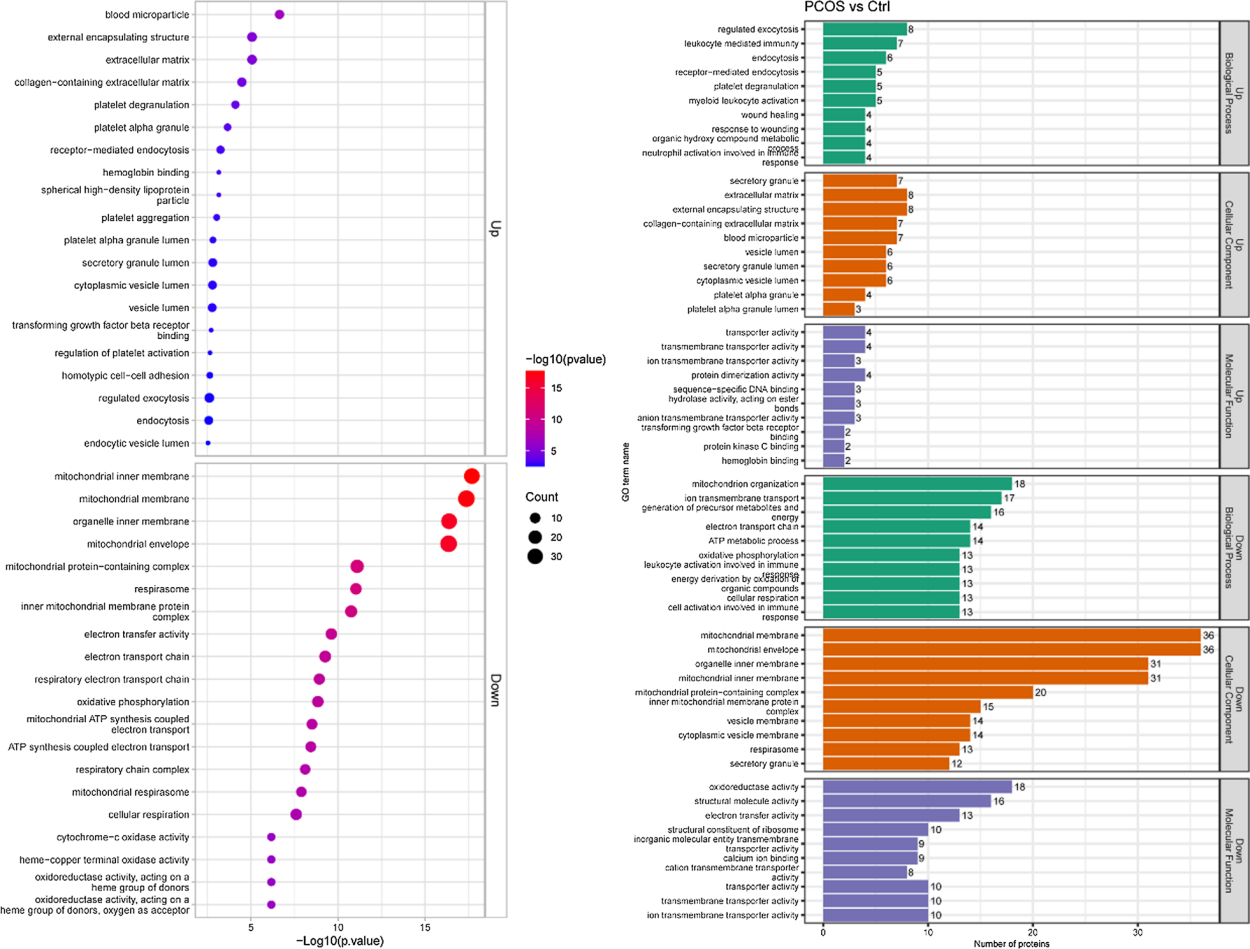
Analysis of differentially expressed protein data between PCOS and CON. **a** Protein quantification results differential analysis statistics are presented in the form of volcano plots. Lower panel a volcano plot generated for two group comparison. It is a visualized graph by plotting "" log2 fold change "" on the x-axis versus; **b** The heat map (P < 0.05)

The differentially expressed proteins were screened and evaluated (Use a change horizon of a substantial increase of > 0.05 and a differential expression ratio > 1.5), and the differential multiple less than -0.67 and p.value < 0.05 as the degree of modification required for meaningful downregulation,the contents of 144 proteins in patients with PCOS were significantly different from those in controls. Details of these proteins are shown in Table 3. Of these proteins, 105 are down-regulated and 39 are up-regulated (Fig. 6a). The heat map (Fig. 6b) also shows the level of these proteins' expression, which is noticeably aggregating in samples from the PCOS and the CON groups.

**Table 3:** Differentially Expressed Metabolites between PCOS and CON

Metabolites	VIP	P-value	LOG_FOLDCHANGE
SHexCer(d39:2)	2.036696885	0.003232657	− 0.587828096
SHexCer(d38:1)	2.031323072	0.047821622	− 0.583723626
PC(20:3e/26:4)	1.930456378	0.002304217	− 0.801315319
PC(14:1e/21:2)	2.02579625	0.049412389	− 1.114659166
PE(16:0/18:1)	1.596845283	0.033344821	0.789111935
PC(16:2e/22:6)	1.508021762	0.023950445	− 1.096931641
PE(14:0/22:3)	1.365604755	0.036342855	0.870535337
PE(17:1/17:1)	1.533667435	0.027219901	0.874700246
TAG(14:0/14:0/18:2)	1.178918447	0.038369004	2.644341044
PE(14:1/24:4)	1.259227457	0.043906883	0.903290845
LPA(18:2)	1.667416581	0.046953909	0.893874252
PE(18:0/18:2)	1.460165919	0.026550035	0.950789144
SM(d14:0/23:1)	1.906311444	0.043408886	− 0.515573151
TAG(12:0/16:1/18:1)	1.804482143	0.029010567	2.035001463
SM(d14:1/30:2)	1.97041388	0.030017463	− 0.57107321
DAG(16:0/16:0)	1.619683925	0.032340506	1.235978374
SM(d14:1/23:1)	1.863754892	0.011902668	− 0.863943677
TAG(14:0/14:0/18:1)	1.897812072	0.027595575	1.842910323
LPC(18:2)	2.030555193	0.011544138	− 0.75404089
PE(20:3/20:3)	1.238573904	0.04525916	0.668340335
TAG(12:0/16:0/18:1)	1.862824165	0.029569476	2.335076593
LPC(22:5)	1.811012258	0.025925893	− 0.593634357
DAG(18:1/18:1)	1.730320285	0.032439327	1.423086939
DAG(16:0/18:1)	1.241612135	0.038333589	0.960209808
LPC(20:4)	2.178208629	0.00176001	− 0.921399262
PE(18:0/22:5)	1.655064796	0.044363733	0.812717152
LPC(22:6)	2.165764176	0.003143105	− 0.79859861
TAG(12:0/16:1/18:2)	1.605741776	0.032086829	1.767289299
DAG(18:1/18:2)	1.117831497	0.046954024	0.855300215
TAG(16:1/16:1/16:1)	1.665668865	0.028281094	1.602642933
FAHFA(22:3/22:4)	1.552990314	0.030294436	1.028892702
PE(22:5e/18:1)	1.657157577	0.025564531	1.232072079
TAG(16:0/16:0/18:1)	1.493641584	0.040676844	1.02766316
TAG(14:0/16:0/18:1)	1.668775885	0.02951231	1.395951672
TAG(16:0/16:1/18:1)	1.468060349	0.043111978	1.078241328
TAG(16:0/16:1/18:2)	1.464200769	0.041091785	1.07966365
TAG(16:0/16:0/16:1)	1.733044538	0.025357913	0.979206153
TAG(12:0/18:2/18:2)	1.727281367	0.033283166	1.89706269
TAG(14:0/18:2/18:2)	1.435462256	0.022925165	0.832663236
TAG(16:2/17:1/17:1)	1.218564562	0.037511005	1.688007585
TAG(14:0/16:0/16:0)	1.986273273	0.024833101	1.868813377
TAG(16:0/16:0/18:2)	1.715570103	0.040437306	0.421163464
TAG(14:0/14:0/20:2)	1.756072963	0.021882398	1.025380714
PE(18:1/18:2)	1.381581045	0.028891554	0.657114004
TAG(16:1/17:1/17:1)	1.469137235	0.041591594	0.459703819
TAG(16:0/16:1/18:3)	1.433228685	0.048140798	1.203184077
TAG(12:1/22:2/22:2)	1.463014061	0.048748029	0.595930683
TAG(12:1/18:0/18:0)	1.80135766	0.01968154	1.152217069
TAG(12:0/12:0/22:1)	1.947272956	0.022650866	1.644359297
TAG(12:0/19:2/19:2)	1.24319542	0.03097402	1.122215671
TAG(12:0/18:1/18:1)	1.721738035	0.012114394	1.677795873
TAG(12:0/12:0/22:2)	1.955325724	0.024601997	2.095190997
TAG(12:0/12:0/14:1)	1.996682462	0.00187642	1.252174694
TAG(12:1/12:1/19:5)	1.470144569	0.036262632	1.049662644
TAG(12:2/12:2/19:4)	1.420365955	0.026123895	1.099251843
TAG(12:3/12:3/21:4)	1.604286235	0.022282638	0.931008228
TAG(15:2/16:5/16:5)	1.691654035	0.020919228	1.927551953
PE(16:0/22:6)	1.227090262	0.048992523	0.634517087
PC(18:1/18:2)	1.309850566	0.021303631	0.461615026
TAG(14:0/16:1/18:1)	1.69782135	0.030956791	1.508267999
PE(18:0/20:3)	1.512106461	0.009168698	0.788752044
PE(16:0/18:2)	1.634900651	0.013572738	0.726539605
LPI(20:4)	1.989505595	0.00262732	− 1.045193951
PE(18:0/22:6)	1.382894015	0.038670735	0.647185268
DAG(16:0/18:2)	1.298355611	0.036510851	0.975354109
HexCer/NS(d18:1/24:1)	1.919861035	0.014585891	− 0.556235945
PC(14:0/18:2)	1.674045538	0.01472253	0.744809956
HexCer/NDS(d23:0/31:1)	1.277482008	0.0417891	0.970501619
GlcADG(20:0/20:0)	1.753121269	0.023931284	− 0.666157874
PC(16:1e/22:4)	1.902957823	0.036609515	− 0.560558333
PE(16:0/22:5)	1.464285786	0.028053894	0.756041839
DAG(18:1/20:4)	1.115497796	0.044924411	0.744365392
TAG(16:0/20:4/22:6)	1.130932029	0.040866827	0.895559295
DGTS(19:0/21:1)	1.455050886	0.045959945	0.665994978
OxPC(18:1/18:0 + 1O(1Cyc))	1.840927036	0.022370549	0.376193063
HexCer/AP(t21:1/14:1)	1.776329301	0.003498639	0.484951386
HexCer/AP(t14:0/21:1)	1.532039091	0.013123571	0.698723519

**Fig. 7:**
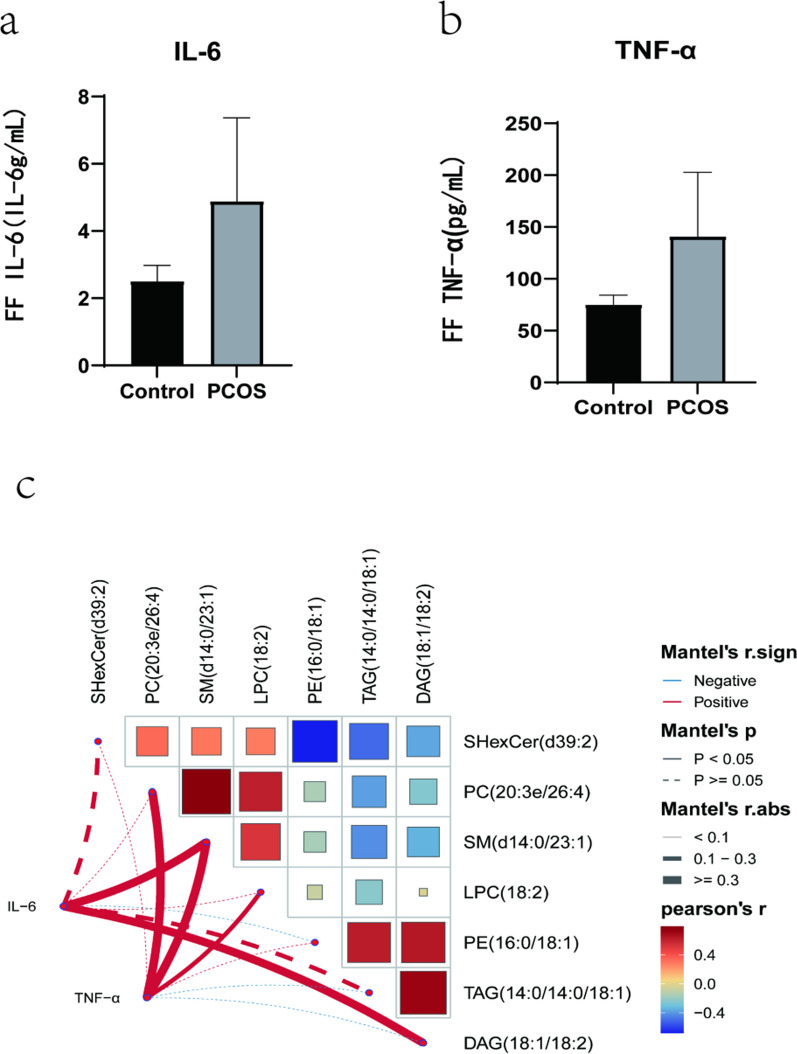
**a** GO functional enrichment analysis; **b** GO functional enrichment analysis. Statistics on the distribution of differentially expressed proteins were performed in GO secondary annotations, including three categories: Biological Process, Cellular Component and Molecular Function, which explain the biological roles of proteins from different perspectives

GO functional enrichment analysis was performed for up-regulated and down-regulated proteins respectively, and the results were shown in Fig. 7a. Terms that up-regulated protein enrichment include blood microparticle, external encapsulating structure, extracellular matrix, collagen − containing extracellular matrix, platelet degranulation, platelet alpha granule, receptor − mediated endocytosis, hemoglobin binding, spherical high-density lipoprotein particle, platelet aggregation, platelet alpha granule lumen and etc. Terms that down-regulated protein enrichment include mitochondrial inner membrane, mitochondrial membrane, organelle inner membrane, mitochondrial envelope, mitochondrial protein-containing complex, respirasome, inner mitochondrial membrane protein complex, electron transfer activity, electron transport chain, respiratory electron transport chain, oxidative phosphorylation, electron transport, mitochondrial ATP synthesis coupled, ATP synthesis coupled electron transport, respiratory chain complex, mitochondrial respirasome, cellular respiration and etc. Statistics on the distribution of differentially expressed proteins were performed in GO secondary annotations, including three categories: Biological Process, Cellular Component and Molecular Function, which explain the biological roles of proteins from different perspectives. GO analysis of up-regulated proteins showed that(Fig. 7b): enriched BP include regulated exocytosis, leukocyte mediated immunity, endocytosis, receptor-mediated endocytosis, platelet degranulation, myeloid leukocyte activation and etc. The down-regulated proteins were enriched in mitochondrion organization, ion transmembrane transport,generation of precursor metabolites and energy, electron transport chain, ATP metabolic process, oxidative phosphorylation, leukocyte activation engaged in immune response, energy derivation by oxidation of organic compounds, cellular respiration,cell activation engaged in immune response.

## Discussion

PCOS,a complex endocrine and metabolic syndrome brought on by the interplay of environmental, genetic, and lifestyle factors,is considered to cause infertility. Clinical features of PCOS include hyperandrogenism, obesity and insulin resistance, accompanied by many long-term complications, such as diabetes, hypertension, hyperlipidemia, cardiovascular diseases and so on. Nowadays, due to technological advances, it is now possible to detect this pathology at an early stage and allow ART treatment to improve the pregnancy rate of PCOS. It has been possible to identify novel proteins and metabolites that are involved in a variety of metabolic and cellular pathways, including the pathogenesis of PCOS, thanks to the advancement of proteomics and metabolomics studies [18]. However, due to the limitation of time and materials, the study of PCOS is still insufficient. In this study,we collected the samples of FF for lipidomic analysis and GCs for proteomic analysis to study the pathogenesis of PCOS.

There were no significant differences in clinical data between the two groups except bLH, LH/FSH, and TT levels.The limited sample size may be the primary cause of these outcomes. There should be a clear clinical difference between the two groups of individuals when the sample size is increased to a specific point.To ensure the freshness of the sample, we selected recent patients from our center, which resulted in a limited sample.Nonetheless, the chosen sample's clinical data showed the fundamental clinical traits of PCOS.The statistical results suggested that there was a typical metabolic syndrome in PCOS patients without statistically significant difference in age and BMI.The results of the expression of proteins and lipids in this investigation are consistent with large sample investigations reported in the literature.

### Changes of lipid metabolism in FF of young women with PCOS

About 70% of PCOS patients have abnormal lipid metabolism, which leads to a series of related clinical manifestations and complications[19]. PCOS affects the patient's oocytes and leads to a low fertilization rate during ART. The FF constitutes the microenvironment for follicle development and oocyte maturation, including secretions from GCS, thecal cells, and oocytes in or around the follicle. Several metabolomic studies of FF have been conducted, including those on carbohydrate, amino acid, and lipid metabolism. In studies that have been done, a number of studies have found that abnormal lipid metabolism involves a number of metabolic pathways such as fatty acid metabolism, glyceripid metabolism, glycerophospholipid metabolism and so on [6, 13].

In this study,we collected FFs of patients and performed a UHPLC-MS–MS analysis. The patients were split into two groups(CON and PCOS) to study differences of each group. The identified lipid species belong to diverse lipid classes,including 53 different types such as ceramides, Lysophophatidylcholine, Phosphatidylcholine, Sphingomyelin, triacylglycerol and etc.

Here, the majority of lipid metabolites were greater in the FF of PCOS group than in the CON group,mainly including TAG,PE,DAG,HexCer-AP. TG usually maintains a high concentration of water during oocyte maturation: during oocyte maturation, lipolysis activity is high, leading to intracellular glycerol biosynthesis. Glycerol kinase expression is also increased in oocytes to facilitate the conversion of glycerol to glycerol 3P and synthesis of TAG to provide energy [20]. In vitro maturation(IVM) of follicles from animal experiments suggests that glycerol and glycerol kinase are essential for follicular development, but that high levels of TG reduce follicular maturation rates. Overweight patients with PCOS often have dyslipidemia, including elevated serum TAG levels [7, 21]. In this study, the BMI of PCOS group has no difference in that of CON group(P > 0.05). Through Spearman correlation analysis, TAG was highly correlated with high-quality embryos and showed a negative correlation trend. The up-regulated hemolytic PE may be due to impaired lysophosphatidyltransferase (LPCAT4) and/or LYPLA1 activity. From this, we hypothesized that the level of TG was increased in the FF of PCOS which was independent of BMI. TG plays a role in oocyte maturation and is an essential component. However, excessive TG levels can affect oocyte development.

Meanwhile, the level of LPC, SM, SHexCer and PC were considerably lower in PCOS FF compared to the CON group,indicating disease-related dysfunction of glycerophospholipid metabolism. Similar trends in PC,LPC decline were discovered in PCOS research using serum and urine [6, 13]. Glycerophospholipids [22], as major structural lipids in mammalian cell membranes, they are essential for controlling transport, signaling, and protein activity. Hemolytic PC is highly associated with apoptosis, inflammation, and glucose regulation.LPC can transmit signals across the cell membrane and has a significant role in glucose regulation [23]. This study also showed that LPC, PC was highly correlated with BMI. In particular, hemolytic PE was positively correlated with No. of oocytes retrieved,MII oocytes, 2PN fertilization, high-quality embryos. All these points to the important role of glycerophospholipid metabolism in ART outcome.

The results found lower levels of SM and SHexCer observed in the FF of the PCOS group, indicating that sphingolipid metabolism is down-regulated in PCOS follicles. The majority of sphingolipids can serve as both structural and signaling molecules for membranes. Sphingolipids have been linked to a number of biological processes, including cell death, proliferation, immunological response, tissue invasion, and metastasis, despite the fact that their involvement in these processes are not fully understood [24]. Glycerophospholipid metabolism is interconnected with sphingolipid metabolism through PC and ceramide synthesis of SM [25]. Y. Shi et al. found that the activation of the PCOS gene YAP (Yes-associated protein) may be impacted by the down-regulated sphingolipid metabolism [26].

### Proteomics of GCs in women with PCOS suggests molecular defects in follicular development

According to the findings of granulosa cell proteomics, 144 proteins were significantly expressed differently in PCOS patients compared to controls. Go functional enrichment analysis revealed that the main enrichment functions of the differentially expressed proteins could be summarized as platelet degranulation, blood coagulation and inflammatory response. PCOS is characterized by a prothrombotic state, often accompanied by lipid abnormalities and platelet dysfunction that induce inflammation. Coagulation and fibrinolytic parameters typically need to be assessed in PCOS patients[27]. Platelets are involved in the formation of corpus luteum and angiogenesis of ovarian granulosa cells, and dysregulation of ovarian angiogenesis can cause abnormal follicular development in PCOS[28, 29].Aye et al. found that in young women with PCOS, acute hypertriglyceridemia caused IR and enhanced platelet activation[30].In this study, the abundant protein changes in platelet degranulation and blood coagulation in patients with PCOS may be the cause of platelet dysfunction, and are also related to IR and inflammation. And we speculated that these differential proteins may be related to follicular development disorder in PCOS patients, thereby participating in the pathogenesis of PCOS.

In addition, inflammation-rich proteins were identified in this analysis, and their association with PCOS has been reported in the literature,such as A1BG,APOC1,AZGP1,CCN4 and etc. [31–34], have a tight connection to the metabolism of lipids, glucose, and obesity, inflammatory response, and insulin resistance,indicating their diagnostic value in PCOS.

Adipocyte factor α-2-Glycoprotein 1 (AZGP1) is a 41 kDa protein that controls insulin sensitivity and glucose lipid metabolism.Several reports suggest that ZAG is associated with inflammation. Liu Y et al. proposed that ZAG may inhibit ERK phosphorylation mediated by TGF-β and inhibit neuroinflammation mediated by TNF-α and IL-6 [35].β-α2-Glycoprotein (ZAG) has been shown to be associated with IR and PCOS [32], respectively. ZAG has been demonstrated to facilitate in vitro fat breakdown and weight loss by enhancing fat loss and reducing TG levels as well as other components of metabolic syndrome (MetS). In a different study, it was observed that IR obese people had lower levels of ZAG mRNA and protein in their adipose tissue compared to lean persons. Lai et al. [36] observed that compared to healthy women, PCOS patients had significantly lower levels of circulating ZAG. However, no research has shown a connection between circulating ZAG levels and the many traits or symptoms used to identify PCOS.

CCN4,known as airfoil-free (Wnt) -induced signaling pathway protein-1 (WISP-1), lately been reported as a novel adipokine obese and insulin resistant people have higher quantities of it in their blood.WISP1 is a downstream target protein of Wnt/ β-catenin. Human adipose-derived stem cells' ability to differentiate into adipocytes can be impacted by the control of the Wnt/-catenin signaling pathway, according to research conducted on animals [37]. WISP1 has been reported to avoid neuronal cell damage and apoptotic degeneration by activating the PI3K/Akt pathway during oxidative damage. Ferrand N [38] found that CCN4 inhibits adipocyte differentiation by inhibiting PPARγ activity. It has been verified that the serum WISP1 value of PCOS group is higher than that of control group. In addition, the patients in obese PCOS subgroup had higher serum WISP1 levels than people who had normal weight and those in obese control subgroups[38]. It is anticipated that WISP1 will emerge as a new therapeutic target for obesity. WISP1 may have a function in the interaction between insulin resistance, inflammation, and obesity. It can be speculated that WISP1 can also be a treatment's therapeutic objective of abnormal lipid metabolism in PCOS patients.

### Relationship between proteomics and lipid metabolomics

The development of oocytes, ovulation, fertilization, embryonic growth and development, and the success of pregnancy are all intimately linked to lipid metabolism.He et al. [39] found that inflammation and abnormal lipid metabolism in PCOS patients have adverse effects on oocyte quality and IVF reproductive outcome.Proteomics analysis showed that the differentially expressed proteins were enriched in inflammation and lipid metabolism.Furthermore, differential lipids and PCOS-related inflammatory markers are tightly associated.Embryo rate in clinical indicators is also correlated with lipid metabolites.It is concluded that the chronic inflammatory state of PCOS patients causes abnormal lipid metabolism, which affects the changes of related proteins and embryo quality in IVF.

## Conclusion

This study showed that significant alterations in glycerolipid, glycerophospholipid, and sphingolipid metabolism have been observed in FF of PCOS patients, and these changes are intimately linked to inflammatory variables.The differential proteins in GCS of PCOS patients are related to lipid metabolism and inflammation.These metabolic dysfunctions are intimately tied to oocyte development in ART. In this experiment, we screened out proteins closely related to lipid metabolism, such as ZAG and WISP1. The limited sample size we examined and the high degree of individual variation in clinical samples may have ramifications for both clinical data and experimental analysis, to sum up. Among the patients we included, there were no statistically significant variations in the results of ART, which is different from the outcomes in a larger trial. This point is the deficiency of the present experiment.

Despite the sample size was limited, these results led us to further investigate the characteristics of the follicular environment related to lipid metabolism and metabolism in PCOS. In clinical practice, it is important to screen lipid metabolic profile and correct chronic inflammatory state before assisted reproductive treatment.Further studies will verify the relationship between differential proteins related to lipid metabolism and follicular development of PCOS, and provide targets and directions for the treatment of PCOS.

## Methods

### Study participants

Patients with PCOS (n = 8) and normal patients with tubal or male factor infertility (n = 8, control group) who underwent IVF were recruited from the Reproductive Medicine Center of Affiliated Hospital of Nanjing University of Chinese Medicine, between May 2022 and January 2023. PCOS was diagnosed using the American Society of Reproductive Medicine's (ASRM) updated criteria. The Nanjing Medical University Ethics Committee authorized our experimental plan(Ethics No. 2022NL-200-03). All the research participants signed the informed consent of the patients. Women with tubal factor infertility met the inclusion criteria for ART. Women who had undergone preimplantation genetic testing (PGT), had a donor oocyte/embryo recipient cycle, diabetes, heart disease, or chronic hypertension were not eligible.

### Collection of follicular fluid and Granulosa cells

All patients were treated with a classical mid-luteal gonadotropin-releasing hormone agonist (GnRHa) long-term regimen.Ovulation was induced by injection of 10,000 IU human chorionic gonadotropin (hCG) on the trigger day. After 36 h, the oocytes were collected by transvaginal B-ultrasound guided puncture. The follicular fluid was extracted from the patient's large follicles (> 18 mm), and the first tube of follicular fluid without blood pollution was collected. The samples were centrifuged at 1500 g for 15 min at room temperature, and the supernatant was removed and stored for future use at – 80 °C. For granulosa cell collection,follicular fluid was centrifuged at 2500 g for 15 min.The precipitate was aspirated and resuspended with percoll, and centrifugation was continued at 2000 g for 20 min to separate blood cells. After the granule cells being collected, they were washed twice with phosphate buffer solution (PBS) for later use.

### Determination of cytokine concentration

ELISA was used to measure the concentrations of TNF-α and IL-6 in follicular fluid.

### Lipid metabolome analysis

#### Metabolites extraction

In an Eppendorf tube, a 100 L aliquot of the sample was transferred, and 480 L of the extract solution (MTBE: methanol1 = 5: 1) was then added. The materials were sonicated for 10 min after being vortexed for 30 s in a cold water bath. Following this, they were centrifuged at 3000 rpm for 15 min at 4 °C (RCF = 900(g), R = 8.6 cm) and then incubated at − 40 °C for 1 h. 350 L of supernatant were moved to a fresh tube and dried at 37 °C in a vacuum concentrator. In order to rehydrate the dried materials, 100 L of a 50% methanol/dichloromethane solution was added. And then the samples were vortexed for 30 s, sonicated in ice water for ten minutes,centrifuged at 13,000 rpm for fifteen minutes at four degrees Celsius. A fresh glass vial was filled with 70 L of supernatant for LC/MS analysis. To create the quality control (QC) samples, 20 L of the supernatant from each sample was combined.

#### LC–MS/MS analysis

The LC–MS/MS studies were carried out using a ultra high performance liquid chromatography-Q exactive orbitrap-mass spectrometry (UPLC-QE-MS) system (1290, Agilent Technologies) and a Kinetex C18 column (2.1 × 100 mm, 1.7 m, Phenomen). Mix mobile phases A and B as required.Per 1000 mL of combined solvent, 50 mL of ammonium formate (10 mmol/L) were added. Set elution gradient feed, autosampler temperature, column temperature, injection volume for positive and negative results, and mobile phase flow rate according to instructions.

Using a QE mass spectrometer and acquisition software (Xcalibur 4.0.27, Thermo), mass spectra were collected in data-dependent acquisition (DDA) mode. In this mode, the acquisition program continuously evaluates the entire scan MS spectrum.

#### Data preprocessing and annotation

Using ProteoWizard's ‘msconvert’ application, the original data files were converted to mzXML files. Peak detection, extraction, alignment, and integration were carried out using the XCMS CentWave algorithm,with minfrac for annotation set to 0.5 and cutoff for annotation set to 0.3. The LipidBlast package, created by R program based on XCMS for spectrum matching, was used to identify lipids.

### Proteomic analysis

#### Protein extraction

GCs samples from 3 normal controls and 3 patients with PCOS were analyzed.Samples of GCs were taken out from − 80 °C, and appropriate 8 M urea cracking solution was added to samples for each group. The samples were placed on ice for 1 h after ice bath ultrasound and vortex fully broken. During this period, vortices were rotated for several seconds every 15 min, and then centrifuged at 12000*g* at 4 °C for 1 h. The supernatant was transferred to a fresh centrifuge tube for protein concentration determination using the Bradford kit.

#### Trypsin digest

In a 56 °C water bath for 25 min, an equal volume of the protein solution was added with dithiothreitol at a concentration of 5 mm. Then, 14 mm of iodoacetamide was added, and it was held at ambient temperature and covered from light for 30 min. Using 25 mm Tris HCl, the samples' urea concentration was reduced to below 2 m. Trypsin was added at a mass ratio of 1 to 100 (protein: trypsin), and the mixture was then enzymatically digested for 16 to 18 h at 37  C.

#### TMT labelling

After being digested, the peptides were desalted using a C18 column and vacuum-freeze dried. According to the TMT kit's operating instructions, peptides were tagged after being dissolved at 0.2 m teab.

#### HPLC fractionation

The XBridge BEH130 C18 column (300 mm 150 mm, 1.7 mm, Waters) was used to grade the peptides using high pH reverse HPLC. These were the operations: 20 mM ammonium formate (pH = 10) made up mobile phase A, and 100% acetonitrile made up mobile phase B, and the gradient of the peptide segment was 3%-41% acetonitrile. The peptides were separated and cyclically collected into 20 components for 73 min, and each component was concentrated into the peptide powder by vacuum and stored at − 20 °C.

#### LC–MS analysis

With 0.1% (v/v) formic acid added, the peptide powder was redissolved, and the EASY-nLC 1200 ultra-high performance liquid system was used to elute it.Formic acid aqueous solution containing 0.1% (v/v) is the mobile phase A, and formic acid acetonitrile solution containing 0.1% is the mobile phase B. Elution gradient: 0 to 5 min, 3% to 5%; 5 to 24 min, 5% to 15%; 24 to 45 min, 15% to 28%; 45 to 52 min, 28% to 38%; 52 to 65 min, 100%; flow rate: 300 nL/min.

The peptides were eluted, ionized, and subjected to series mass spectrometric analyses using Thermo Scientific's Orbitrap Fusion™ Lumos™ Tribrid™. High-resolution Orbitrap was used to detect and analyze the parent ions and secondary fragments of the peptide at 2.1 kV source voltage.Two mass spectrometry analyses' scanning range and resolution were established in accordance with the guidelines, and the data-dependent scanning (DDA) data acquisition mode was used. The steps were as follows: after primary scanning, the top 20 parent ions of the peptide segment with the highest signal intensity were selected, sequentially added to the HCD collision pool, fragmented with 32% fragmentation energy, and then secondary mass spectrometry was carried out. Automatic gain control (AGC) was set to 1.25 E5 and serial mass spectrometry was employed for scanning in order to increase the efficiency of mass spectrometry. To prevent parent ion duplication, the dynamic exclusion time is set at 60 s.

#### Databases search

Secondary mass spectrometry data were extracted with Maxquant (v1.6.14). Retrieval parameters were set: Uniprot-Hom-Sapiens-proteome_up000005640 (2021.06) was used in the database, and Trypsin/P was selected as the technique of enzyme digestion. The number of missing cuts is set to 2; The minimum length of peptide was set to 7 amino acid residues. Methionine oxidation and acetylation of the protein's N-terminal were defined as variable modifications, whereas the alkylation of cysteine was set as a fixed modification. The quantitative technique was set to TPT-6plex, and the FDR for identifying proteins and PSMs was set to 1%.

#### Analysis of clinical data

Data were analyzed with SPSS 26.0 to highlight the fundamental characteristics of the study population. Means and standard deviations (SDs) were used to express continuous variables with normally or nearly normally distributed distribution.

## Data Availability

The data underlying this article are available in the article and in its online supplementary material. Portions of the data from this article will be shared upon reasonable request from the corresponding author.
